# Vinculin Force Sensor Detects Tumor-Osteocyte Interactions

**DOI:** 10.1038/s41598-019-42132-x

**Published:** 2019-04-04

**Authors:** Fangjia Li, Andy Chen, Andrew Reeser, Yue Wang, Yao Fan, Shengzhi Liu, Xinyu Zhao, Rahul Prakash, Divya Kota, Bai-Yan Li, Hiroki Yokota, Jing Liu

**Affiliations:** 1 Department of Physics, Indiana University Purdue University Indianapolis, Indianapolis, IN 46202 USA; 2 Department of Biomedical Engineering, Indiana University Purdue University Indianapolis, Indianapolis, IN 46202 USA; 3 Department of Pharmacology, School of Pharmacy, Harbin Medical University, Harbin, 150081 China; 4 Peking Union Medical College Hospital, Chinese Academy of Medical Sciences, Beijing, 100730 China; 5 Department of Nanoscience and Nanoengineering, South Dakota School of Mines and Technology, Rapid City, SD 57701 USA

## Abstract

This study utilized a Förster resonance energy transfer (FRET)-based molecular tension sensor and live cell imaging to evaluate the effect of osteocytes, a mechanosensitive bone cell, on the migratory behavior of tumor cells. Two cell lines derived from MDA-MB-231 breast cancer cells were transfected with the vinculin tension sensor to quantitatively evaluate the force in focal adhesions of the tumor cell. Tumor cells treated with MLO-A5 osteocyte-conditioned media (CM) decreased the tensile forces in their focal adhesions and decreased their migratory potential. Tumor cells treated with media derived from MLO-A5 cells exposed to fluid flow-driven shear stress (FFCM) increased the tensile forces and increased migratory potential. Focal adhesion tension in tumor cells was also affected by distance from MLO-A5 cells when the two cells were co-cultured, where tumor cells close to MLO-A5 cells exhibited lower tension and decreased cell motility. Overall, this study demonstrates that focal adhesion tension is involved in altered migratory potential of tumor cells, and tumor-osteocyte interactions decrease the tension and motility of tumor cells.

## Introduction

Breast cancer is one of the most common cancers among women, and almost 30% of primary breast tumors are reported to metastasize to other organs^1^. Along with the brain and lungs, bone is one of the most frequent sites of metastasis^2,3^. While the exact reasons for the high risk of bone metastasis is not well understood, we have previously reported that osteocytes may act as an attractor of migratory breast cancer cells via matrix proteins such as collagen^4,5^. Osteocytes are the most abundant type of bone cells in bone^6^. A comprehensive understanding of the mechanisms behind tumor-osteocyte interactions is critical for developing novel options for the treatment of bone metastasis associated with breast cancer.

In this study, we investigated the effects of osteocytes on migratory behaviors of breast cancer cells. In particular, we addressed the question: Does mechanical stimulation to osteocytes alter their effects on molecular machinery and migratory capacity of breast cancer cells? Osteocytes are mechano-sensors, which can propagate loading-driven signaling and activate differentiation of bone-forming osteoblasts^7–9^. We hypothesized that in the presence and absence of mechanical stimulation, osteocytes interact differently with breast cancer cells via molecular machinery at focal adhesions. To test this hypothesis, we employed a molecular tension sensor as well as live cell imaging to investigate the force dynamics of the focal adhesion during the regulatory migration behaviors.

The vinculin tension sensor, in which a Förster resonance energy transfer (FRET) based tension sensor module (TSMod) was inserted between the head and tail domains of the vinculin protein, was first introduced by Grashoff *et al*.^10^. The TSMod includes an elastic peptide linker of 40 amino acids and two fluorophores (mTFP1 and Venus) on each end, in which extension of the linker changes FRET between fluorescent proteins. This vinculin tension senor is recruited to focal adhesions (FAs) which connect integrin and the actin filaments^11,12^ and are sensitive to mechanical stimulation and induce signaling for cell migration through extracellular matrices^13^. This biosensor could help us to understand many developmental and pathophysiological processes from a mechanical point of view. Especially in tumor cells, focal adhesions are an important means of force transmission and reception with the extracellular matrix^14,15^.

To understand the forces on focal adhesions of tumor cells interacting with osteocytes, we conducted FRET analysis using a vinculin tension sensor transfected into the tumor cells. In this study, we employed MLO-A5 osteocytes with TMD and BMD breast cancer cells (derived from MDA-MB-231 cells). Tumor cells were transfected with the vinculin tension sensors. Post-transfection, the cells were treated in different conditions, and FRET efficiency of the tensions sensor was evaluated. To evaluate migratory behaviors, scratch assays were performed on the treated cultures. Cell tracking under various treatment conditions with the IncuCyte ZOOM live-cell imaging platform was used to evaluate osteocyte-induced migrationary behaviors of the tumor cell. The results indicate that migratory behaviors of tumor cells are closely linked to tensile forces at focal adhesions, and in response to mechanical stimulation osteocytes toggle their regulation of migration in tumor cells. This study suggests a critical role for mechanotransduction of bone in tumor metastasis and a novel target of interactions in bone metastatic tumor cells.

## Results

### Characterization of vinculin tension sensor (VinTS) and tailless probe (VinTL)

**Figure 1:**
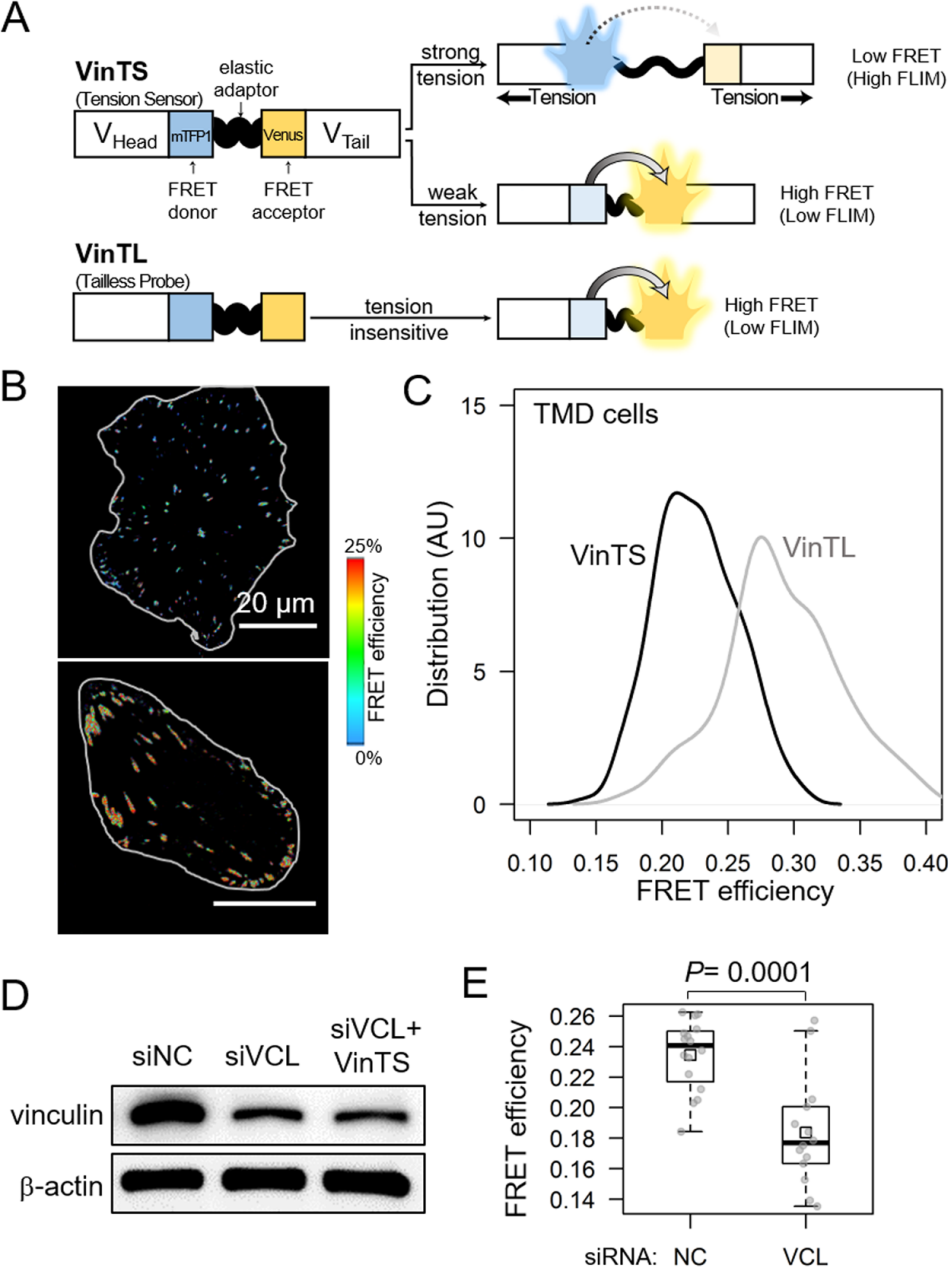
Characterization of the vinculin tension sensor. (**A**) Schematic illustration of VinTS (vinculin tension sensor) and VinTL (tailless probe as a negative control). (**B**) Representative images for a comparison of FRET efficiency between VinTS and Vin TL. (**C**) Distribution of FRET efficiency for VinTS and VinTL. (**D**) Partial silencing of vinculin with siRNA. Of note, siNC = non-specific control siRNA, siVCL = vinculin siRNA, and siVCL + VinTS = vinculin siRNA and vinculin tension sensor. (**E**) Reduction in FRET efficiency by siRNA specific to vinculin.

In this study, we employed two molecular probes, VinTS (vinculin tension sensor) and VinTL (vinculin tailless) probes (Fig. 1A) to demonstrate and validate the function of the tension sensing in focal adhesion. The VinTS is a tension sensitive sensor that consists of the head and tail domains of vinculin with the elastic FRET module inserted between them (Fig. 1A), and the sensor exhibits low(high) FRET efficiency when it undergoes high (low) tensions (Fig. 1A,B); the FRET map of the TMD cell transfected with the VinTS probe has shown low and varying FRET efficiency for individual focal adhesion sites (Fig. 1B). As a comparison, the VinTL probe is missing the tail domain and thus insensitive to tension (high FRET), and the FRET map shows a higher FRET efficiency (Fig. 1B,C). In addition, the VinTS probe successfully reports the reduced forces (high FRET) in focal adhesion when the cell is treated by ML-7, which is a myosin II inhibitor and leads to less tensile forces in cells^16,17^. As an opposite, the cell exhibits low FRET efficiency (higher forces) when cells are treated by Calyculin A, a myosin II activator that stimulate the tension (Suppl. Fig. S1)^17,18^. In our work, the force experienced by the VinTS in focal adhesions is calculated based on the FRET efficiency according to the calibration curve reported in ref.^10^ (Materials and Methods).

Vinculin is known to participate in cellular migration, and reduction in vinculin in tumor cells is reported to promote migratory behaviors^19,20^ (Suppl. Fig. S2). To characterize the vinculin tension sensor, we evaluated FRET efficiency in TMD tumor cells with reduced vinculin expression. When vinculin was partially silenced in TMD cells by RNA interference (Fig. 1D), a significant reduction in FRET efficiency (from 24% to 18%) was observed (Fig. 1E) for the VinTS probe, and the expression of the VinTS did not affect the silence of the vinculin (Fig. 1D). Hereafter, we mainly used the VinTS probe and evaluated migratory behaviors of tumor cells in the presence and absence of MLO-A5 osteocyte cells or their conditioned medium.

### Reduction in tensile force by MLO-A5 osteocyte-derived conditioned medium

**Figure 2:**
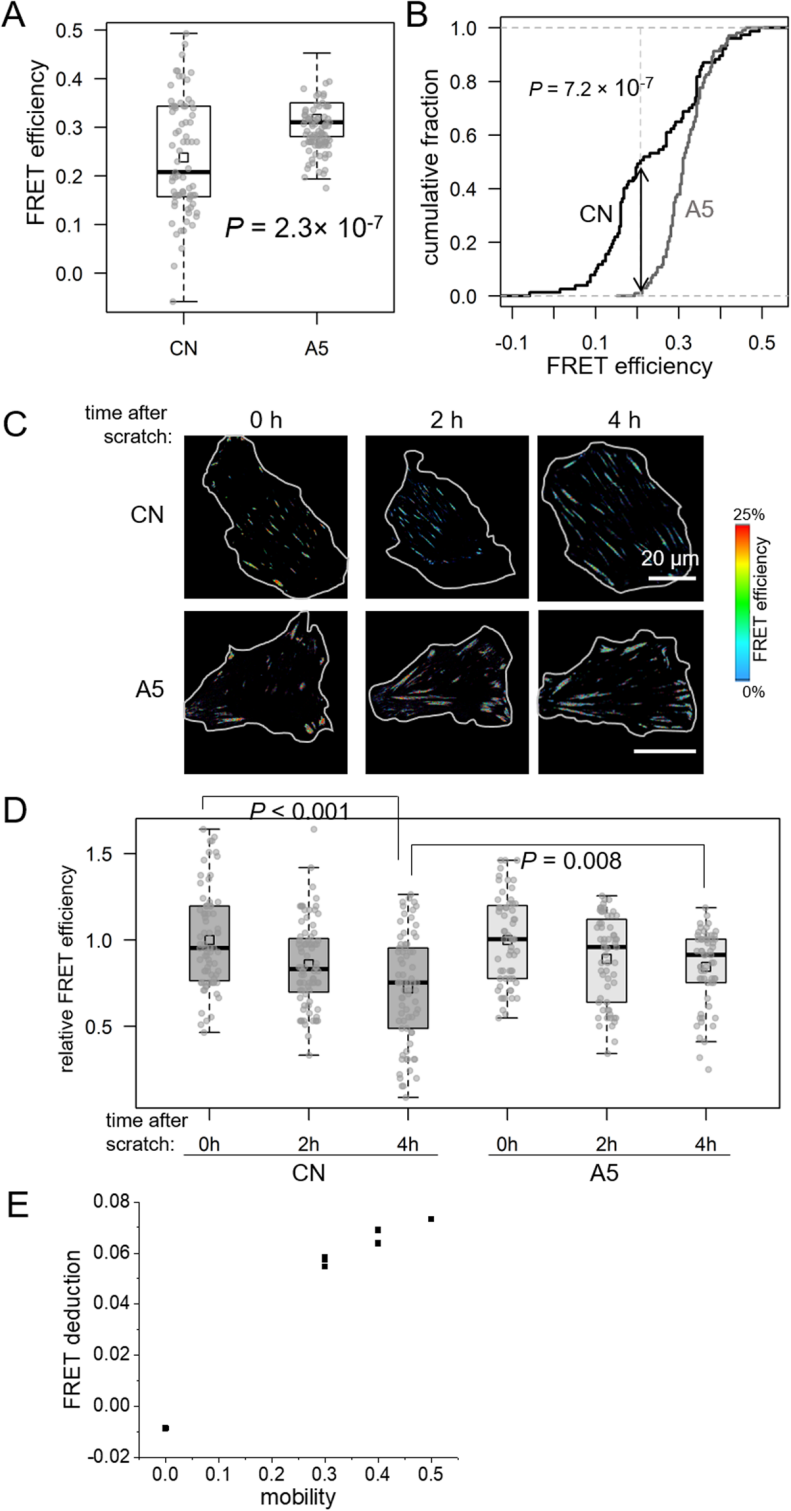
Effects of MLO-A5 osteocyte-derived conditioned medium (CM) on FRET efficiency in TMD cells. (**A**,**B**) Increase in FRET efficiency by CM. (**C**) Representative images, showing FRET efficiency in a scratch assay in the control medium (CN) and CM. (**D**) CM-driven increase in FRET efficiency in a scratch assay. (**E**) Correlation between the deduction of the FRET efficiency and the cellular mobility.

Using TMD tumor cells transfected with VinTS, we first examined the effects of MLO-A5 osteocyte conditioned medium (CM) on FRET efficiency and tensile forces. Incubation of TMD cells in CM significantly increased the mean value of the FRET efficiency (10% on average) and shrink the standard deviation of the statistics (Fig. 2A); of note, 10% change in FRET efficiency is estimated to correspond to a force deduction of 0.76 pN. We also used the Kolmogorov-Smirnov test to compare the distributions of FRET efficiency in control and CM-treated TMD cells and found they are different (Fig. 2B). Furthermore, we also monitored the migration behavior of a single cell for four hours on a wound scratch, where the FLIM image of the cell was continually recorded every two hours. It has been found that the tumor cell on the edge of the scratch tended to migrate to the freshly scratched spaces, and the migrating TMD cells progressively decreased FRET efficiency. Such a tendency was observed in both control medium and CM, while the FRET efficiency decrease of TMD cells in CM was significantly smaller than that in the control medium (Fig. 2C,D). In addition, we also correlate the FRET efficiency with the cell mobility, which was derived from the cell morphology and positions on the scratched area; and it was found that the tensile force increases approximately linearly as motility increases (Fig. 2E). Collectively, the results indicate that osteocytes can alter the behavior of tumor cells through their culture medium.

### Linkage of tensile force to migratory behaviors

**Figure 3:**
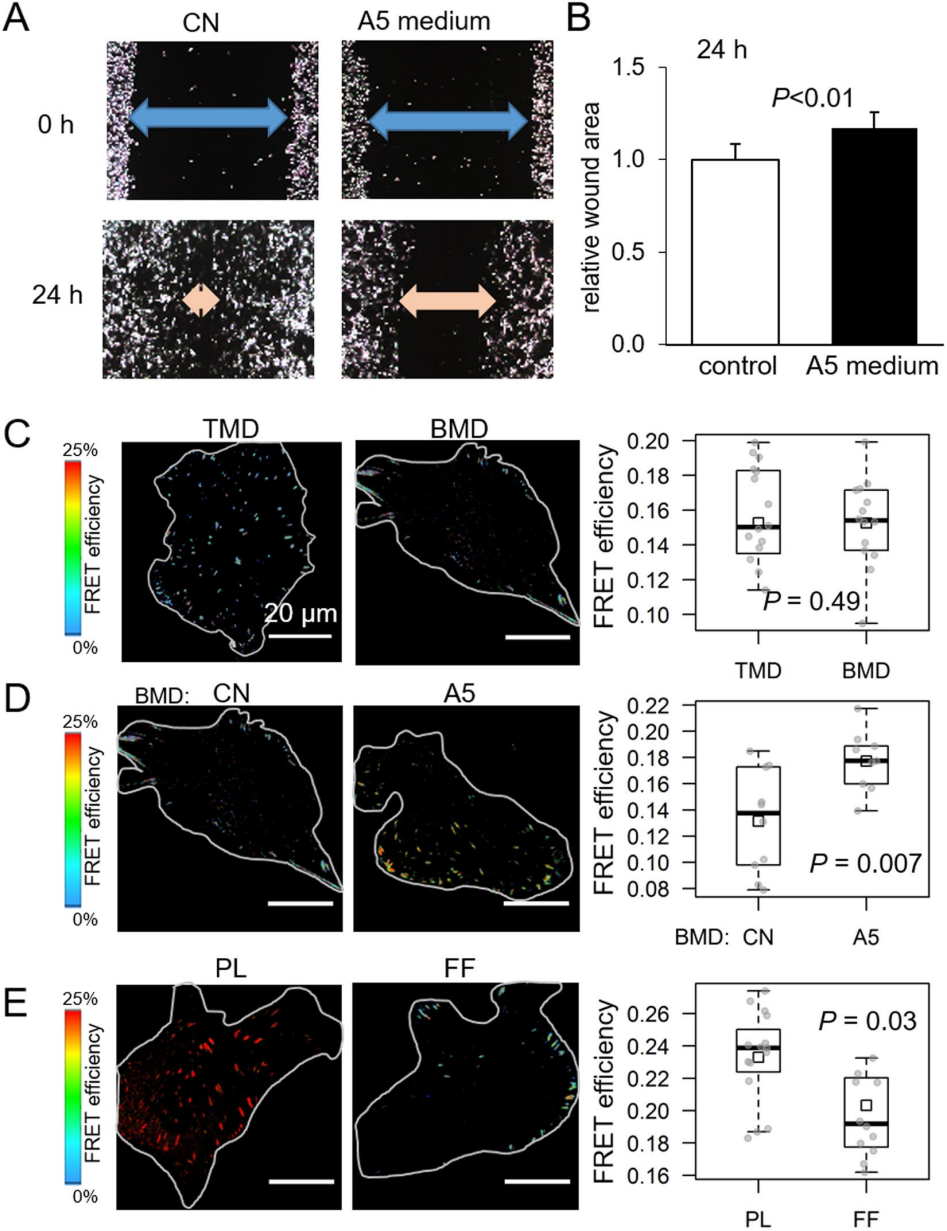
Differential effects of MLO-A5 osteocyte conditioned medium (CM) and fluid flow-treated conditioned medium (FFCM). Of note, CN = control, and PL = placebo in a flow chamber without fluid flow treatment. (**A**,**B**) CM-driven inhibition of cellular migration of TMD cells in a scratch assay. (**C**) Comparable FRET efficiency of TMD cells and BMD cells. (**D**) CM-driven increase in FRET efficiency in BMD cells. (**E**) FFCM-driven decrease in FRET efficiency in BMD cells.

We next examined the effect of CM on cellular migration. Migratory behaviors of TMD cells were first evaluated by a wound healing scratch assay in control medium and CM. Consistent with the CM-driven increase in FRET efficiency, CM inhibited cellular migration of TMD cells (Fig. 3A,B). Besides TMD tumor cells, we also employed BMD tumor cells, a bone metastasis-derived clone of MDA-MB-231 breast cancer cells that are less migratory than TMD cells^21^. The result showed that no significant difference was observed in baseline FRET efficiencies in BMD and TMD cells (Fig. 3C), and CM also increased FRET efficiency in BMD cells (Fig. 3D).

### Differential effects of fluid flow-treated CM on tumor behavior

Osteocytes are known to be mechano-sensors in bone matrix and induce loading-driven bone formation^8^. We tested whether fluid flow treatment of osteocytes may alter their conditioned medium’s effects on the migratory behaviors of tumor cells. Fluid flow-treated conditioned medium (FFCM) was prepared by applying oscillatory fluid flow to A5 osteocyte cells (1 Hz for 1 h with 0.8 Pa). The response of TMD cells to FFCM was opposite to their response to CM, and the TMD cells presented a decrease in FRET efficiency and an increase in tensile force (Fig. 3E). The reduction of FRET efficiency from 24% to 19% corresponds to an increase in tensile force from 2.7 to 3.1 pN.

### Effects of collagen treatment and Snail overexpression on migration of tumor cells

**Figure 4:**
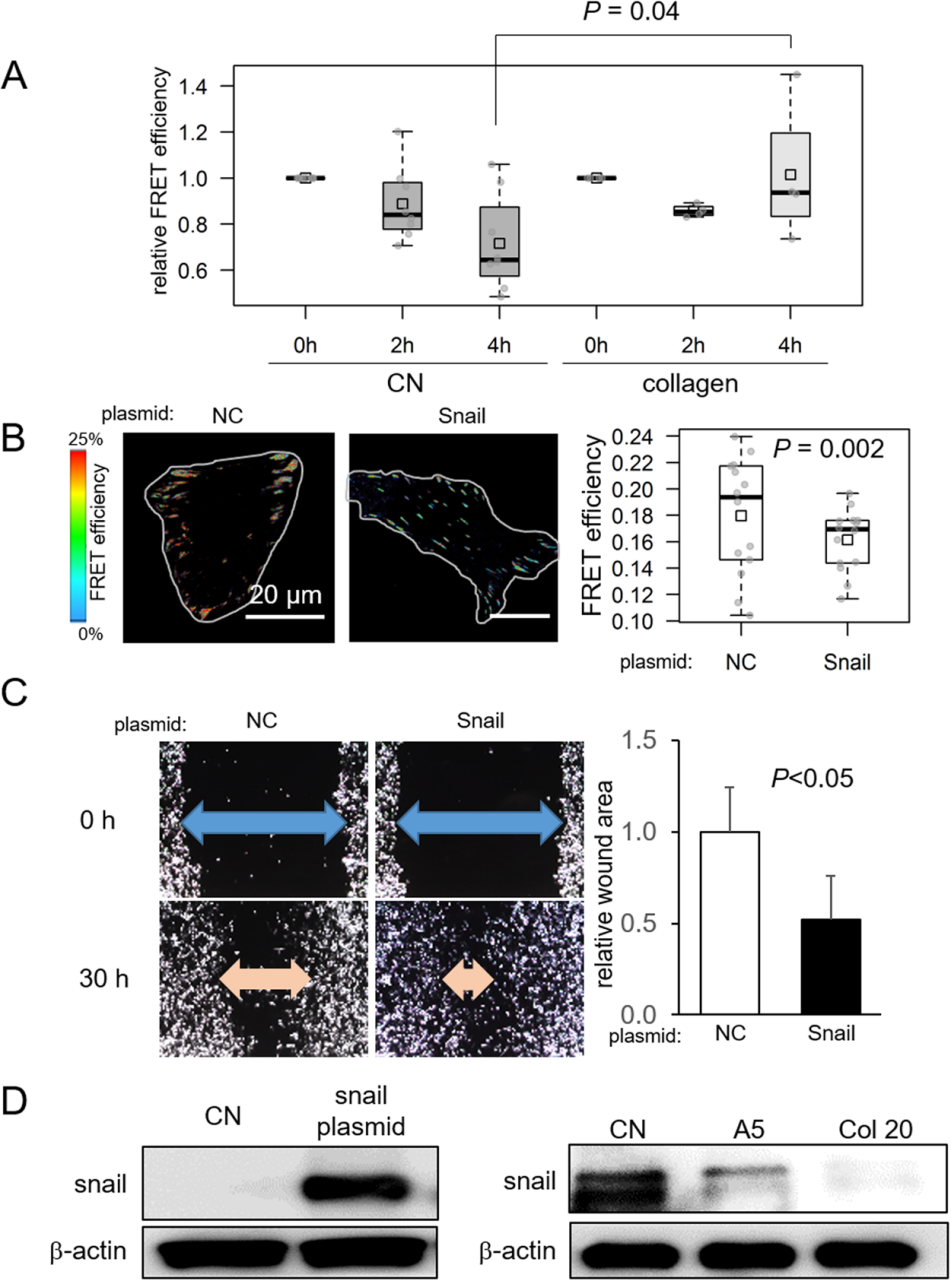
Effects of collagen and Snail plasmids on cellular behaviors of TMD cells. (**A**) Increase in FRET efficiency by treatment with 10 μg/ml collagen. (**B**) Decrease in FRET efficiency by overexpression of Snail. (**C**) Increase in cellular migration by Snail plasmids in the scratch assay. (**D**) Upregulation of Snail by transfection of Snail plasmids, and downregulation of Snail by CM as well as treatment with 20 μg/ml collagen.

Our previous studies indicated that collagen-treated medium causes breast cancer cells to undergo suppression of migratory behaviors similar to that caused by CM^4^. To determine if collagen-treated medium may cause detectable alterations in cellular forces, a time-course scratch assay was conducted. Like CM, the result revealed that the change in FRET efficiency by the collagen-treated medium was significantly smaller than its reduction in a control medium (Fig. 4A). Furthermore, overexpression of Snail resulted in a decrease in FRET efficiency, indicating an increase in tensile force (Fig. 4B). It also promoted cellular migration of TMD cells (Fig. 4C,D). Consistent with the observed migratory behaviors and the stimulatory role of Snail in cellular migration, both CM and collagen treatment downregulated Snail (Fig. 4D).

### Tumor-osteocyte interactions through live cell imaging

**Figure 5:**
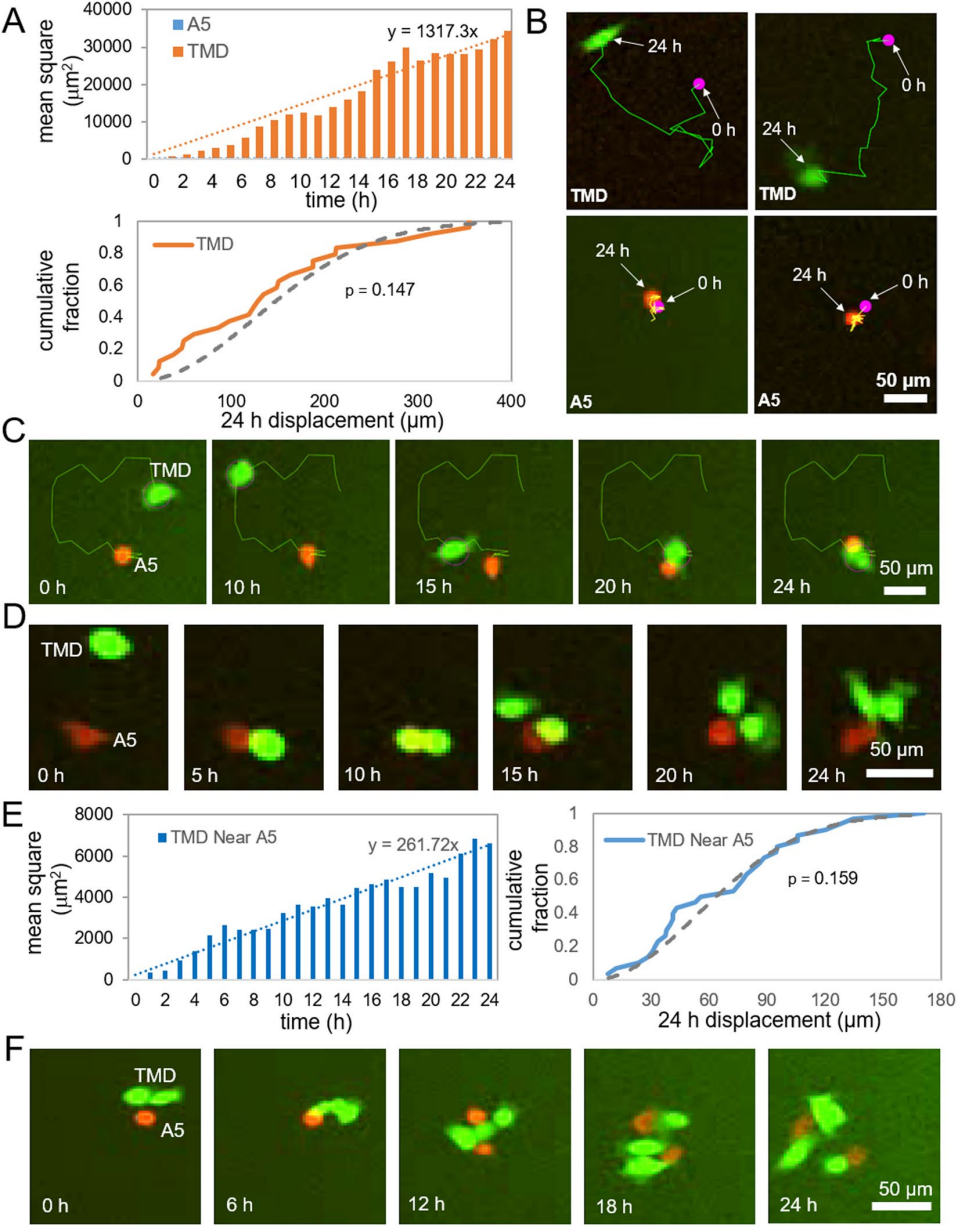
IncuCyte imaging of migratory behaviors of A5 osteocyte cells and TMD cells. (**A**) Mean-squared migratory distances, and the cumulative distribution of migratory distances at 24 h with Rayleigh distribution in a dotted curve. (**B**) Representative migratory trajectories of TMD cells and A5 osteocyte cells from 0 h to 24 h. (**C**,**D**) Sample trajectories of a single TMD cell, moving towards a single A5 osteocyte cell. (**E**) Mean-squared migratory distances, and the cumulative distribution of migratory distances of TMD cells, locating within 120 µm of A5 osteocyte cells. The dotted curve in the cumulative distribution represents Rayleigh distribution. (**F**) Sample trajectories of two TMD cells interacting with a single A5 osteocyte cell.

In the experiments so far, we demonstrated the role of tumor-osteocyte interactions in migratory behaviors with CM and FFCM. Using IncuCyte live cell imaging, we next evaluated the interaction of individual tumor cells with A5 osteocyte cells. From a set of time lapse images of individual TMD cells as well as A5 osteocyte cells, we traced cell trajectories for 24 h with an increment of 1 h (Fig. 5A,B). The increase in mean-squared displacement (MSD) of TMD cells from their initial position in the absence of A5 osteocyte cells was almost linearly proportional to time, and the cumulative distribution of displacement (0 h to 24 h) was indistinguishable from the Rayleigh distribution (*p* = 0.147), indicating that migration of tumor cells can be approximated by a random-walk coil^22^. When a single A5 osteocyte cell was in the vicinity of a TMD cell, the tumor cell was frequently attracted to the osteocyte (Fig. 5C,D). While the overall character of mean-squared distances and end-to-end distance distributions were the same to those without A5 osteocytes in proximity, the slope of the MSD to time, which indicates the diffusive mobility, was reduced 5-fold (from 1,317 to 262).

### Higher FRET efficiency (lower tensile force) with osteocytes in proximity

**Figure 6:**
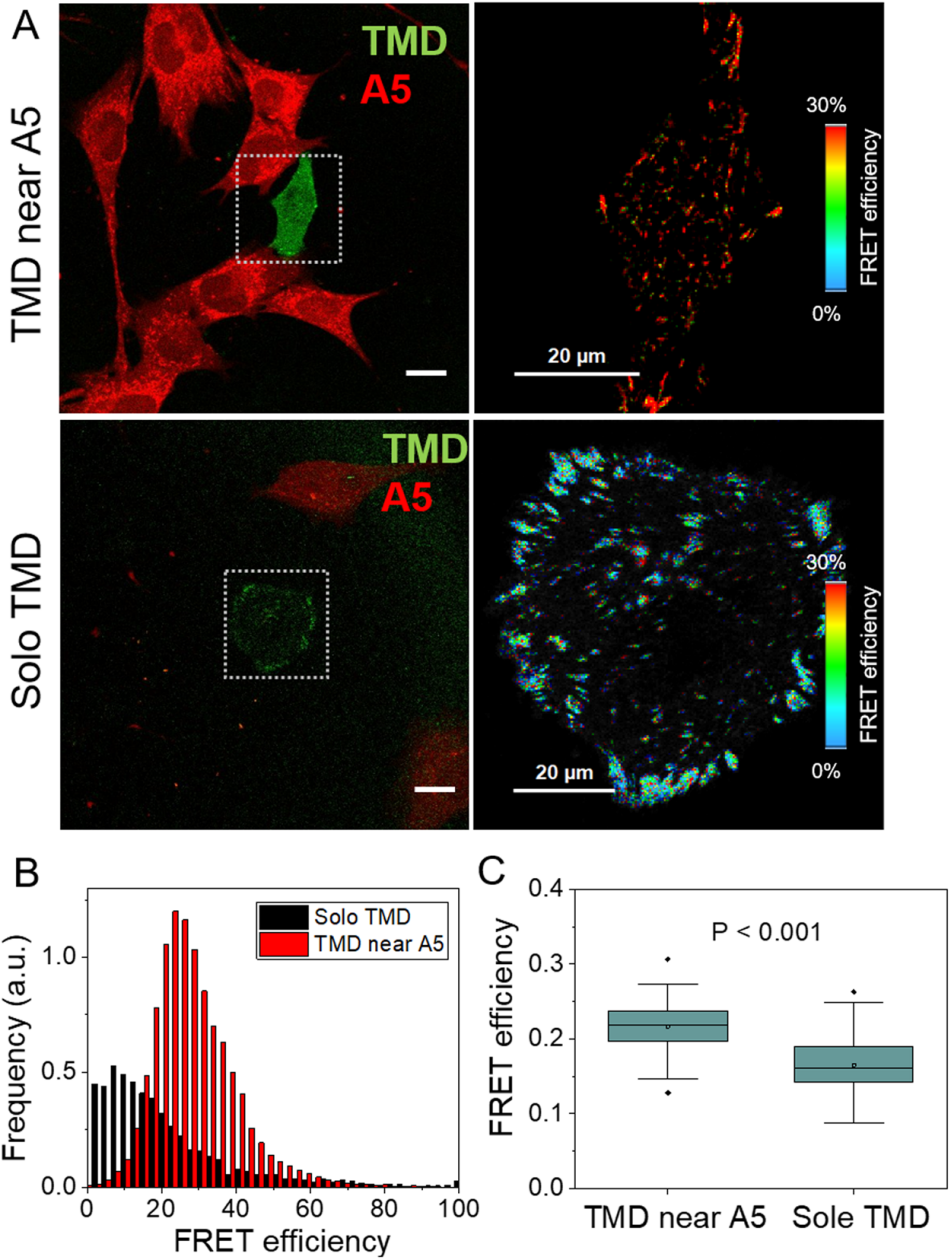
Effects of A5 osteocyte cells on migratory trajectories of TMD cells. (**A**) Representative images of TMD cells in red and A5 osteocyte cells in green, and the color-coded FRET efficiency. The top images correspond to two cells in close proximity, while the bottom images in separation. (**B**) Distribution of FRET efficiency of TMD cells with A5 osteocyte cells in proximity and in separation. (**C**) Comparison of FRET efficiencies with A5 osteocyte cells in proximity and separation (100 µm or more distant).

Live cell imaging analysis of TMD and A5 cell movement support the notion that migratory behaviors of tumor cells were significant altered by adjacent A5 osteocyte cells. To see the proximity effect on focal adhesion tension, TMD cells and A5 osteocyte cells were co-cultured, and the FRET efficiency of TMD cells in the presence and absence of neighboring A5 osteocyte cells were determined (Fig. 6A). The results revealed that compared to isolated TMD cells, TMD cells with nearby A5 osteocyte cells presented higher FRET efficiency (lower tensile forces) (Fig. 6B,C). Numerical simulation was conducted to generate migratory trajectories of tumor cells using a random-walk model (Suppl. Fig. S3).

## Discussion

By measuring focal adhesion forces with a vinculin tension sensor and analyzing migratory behavior with real-time live cell imaging, this study revealed that metastatic tumor cells are attracted to osteocytes, and this attraction is reversed by applying mechanical stimulation to osteocytes. As part of the molecular machinery regulating cellular motility, vinculin is reported to act as an inhibitor of tumor migration^19^. In our experiment, a partial silencing of vinculin by RNA interference led to a significant decrease in FRET efficiency associated with an increase in the generation of tensile force that promotes migratory capacity of tumor cells; the result confirms that the migration of the cell is stimulated by the depletion of the vinculin. Tumor cells were exposed to osteocyte-derived CM or collagen-treated medium, FRET efficiency was elevated along with a decrease in migratory capacity. When they were cultured in fluid flow-treated CM or transfected with Snail plasmid, however, the outcome was opposite, the migratory capacity of tumor cells was enhanced, and the decreased FRET efficiency is accompanied. Collectively, this study suggests a critical role for osteocytes in modulating the migratory behaviors of tumor cells, acting as both a stimulator as well as an inhibitor depending on the biophysical condition of the bone microenvironment.

**Figure 7:**
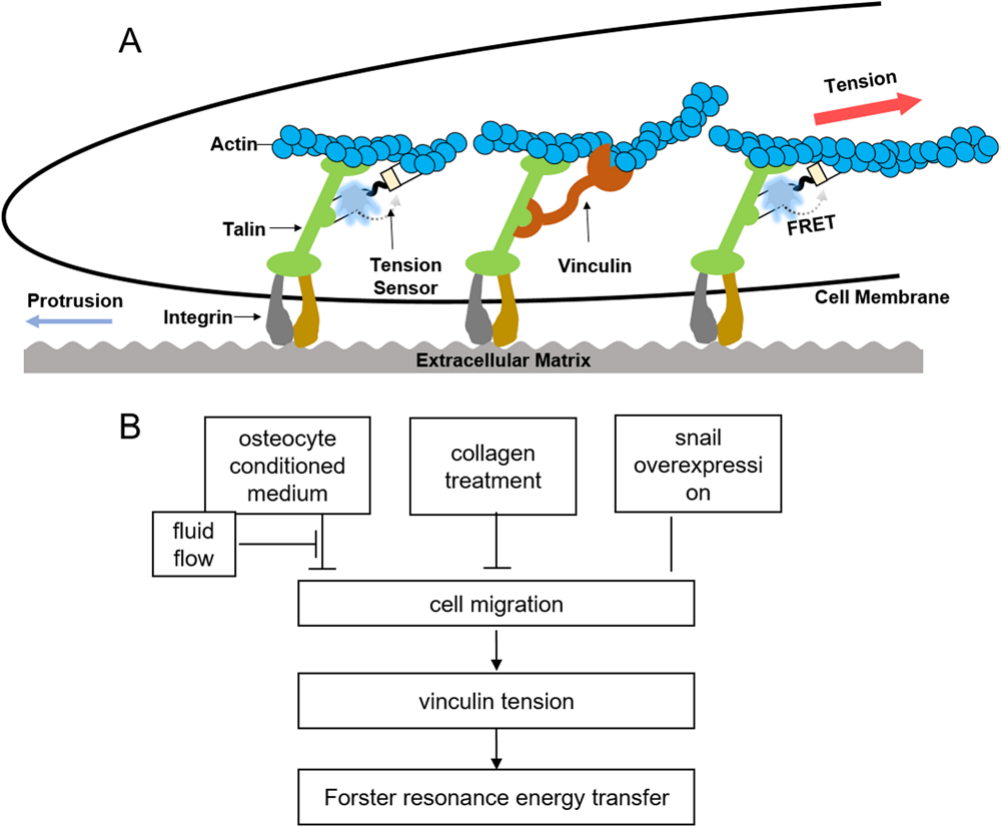
(**A**) Schematic illustration of a migratory tumor cell interacting with extracellular matrix. A FRET sensor, VinTS, reports the tension applied on the focal adhesion sites that is reflected in a cell migratory. (**B**) Proposed mechanism in cellular migration and vinculin tension. While the CM and collagen inhibit the cell migration, the FFCM and the snail overexpression had an opposite effect on cell migration. The force dynamics during these processes is reported by the vinculin tension sensor through FRET.

The vinculin tension sensor employed in this study is a powerful tool to evaluate the linkage between molecular forces and cellular motility. The measurements derived from the FRET-based probe were consistent with the migratory trajectories of tumor cells observed by IncuCyte live cell imaging. When osteocytes were positioned close to a tumor cell, the tumor cell exhibited high FRET efficiency, indicating that proximity to osteocytes lead to generation of decreased tensile forces and low cellular motility. Since FRET efficiency is highly sensitive to the distance between a pair of donor and acceptor domains across the elastic adaptor, it was possible to estimate induction of molecular forces as small as 1 pN. In the schematic illustration in Fig. 7A, an actively migrating tumor cell is shown to be under tensile force that is transmitted to the intramolecular elastic adaptor of the vinculin tension sensor via integrin, talin, and actin filaments^23^.

Using IncuCyte live cell imaging, we evaluated individual interactions of a single tumor cell with a single osteocyte cell. Although the shapes of the mean-squared distances and end-to-end distance distribution were nearly the same in the presence and absence of nearby A5 osteocytes, the slope of the mean-squared distances was significantly reduced when A5 osteocytes were present. The slope is proportional to the diffusion coefficient in a random walk model^24^. Furthermore, the FRET imaging analysis revealed that TMD cells with nearby A5 osteocyte cells presented lower tensile forces than TMD cells without A5 neighbors. Collectively, live cell imaging and the FRET-based tension sensor demonstrate that direct tumor-osteocyte interactions decrease the tensile forces and motility of tumor cells (Fig. 7B).

While osteocyte-derived CM and collagen-treated medium was used to mimic part of the bone microenvironment, the results herein demonstrate the role of bone mechanotransduction in the migration and colonization of breast cancer cells. This study also suggests the importance of molecular dynamics at focal adhesions where vinculin is actively engaged. It is not completely clear, however, whether mechanical stimulation such as fluid flow can protect bone from osteolysis associated with tumor metastasis. Fluid flow-driven migration of tumor cells may reduce the population of tumor cells in the bone microenvironment and prevent their colonization in bone. However, it may also promote invasiveness of tumor cells by upregulating Snail that induces epithelial to mesenchymal transition^25^. Further analysis is necessary to understand the holistic view of tumor-osteocyte interactions and its implication on clinical practice.

In this study, we evaluated tumor-osteocyte interactions using the vinculin tension sensor and real-time live cell imaging. While these techniques were useful for our understanding of the bone microenvironment at the molecular and cellular levels, the study is not free from limitations. While the vinculin sensor is sensitive to tensile forces, tumor migration is an orchestrated process in which many motor and structural proteins are involved at focal adhesions and in the cytoplasm and nucleus. Usage of other techniques such as traction force microscopy may help capture a more comprehensive picture of the molecular machinery^26^. Second, the experiments herein employed mouse osteocytes with human breast cancer cells. The observed interactions may differ depending on the types of osteocyte and tumor cells^27^. Lastly, besides collagen and Snail, other proteins in the extracellular matrix or secretory factors such as integrin, fibronection, and osteopontin may also play an important role in tumor-osteocyte interactions^4,28^. In conclusion, this study demonstrates that a vinculin tension sensor can be used to measure cell tensile forces to evaluate the behavior of tumor cells in tumor-osteocyte interactions. The result herein might contribute not only to our basic understanding of tumor growth and migration in the bone microenvironment but also toward developing novel therapies to prevent bone metastasis associated with breast cancer.

## Materials and Methods

### Cell culture

MDA-MB-231 human breast cancer cell-derived cell lines, TMD cells and BMD cells^29^, were grown in DMEM (Corning, Inc., Corning, NY, USA), and MLO-A5 osteocyte-like cells were grown in αMEM (Gibco, Carlsbad, CA, USA)^30^. For TMD and BMD cells, the culture media was completed with 10% fetal bovine serum (FBS) and antibiotics (50 units/ml penicillin, and 50 µg/ml streptomycin; Life Technologies, Carlsbad, CA, USA). For MLO-A5 cells, the culture media contained 5% FBS and 5% fetal calf serum with antibiotics. Cells were maintained at 37 °C with 5% CO_2_.

### IncuCyte imaging

To observe migratory behaviors of TMD cells with MLO-A5 osteocyte cells, IncuCyte ZOOM real-time imaging microscope was used to image cells every hour with fluorescent staining (Incucyte CytoLight Green and Red, Essen Bioscience, Michigan, USA).

### FRET imaging

The fluorescence lifetime images were acquired by a custom-made fluorescence lifetime imaging microscope built on a laser scanning confocal microscope (FluoView 1000, Olympus). A picosecond pulsed laser with the wavelength of 450 nm (LDH-D-C-450, Picoquant) was coupled with the laser scanning module, and the excited fluorescent signal was filtered by a band-pass filter 490-40 (ET490/40 × , Chroma) before entering a photon counting detector (PD-100-CTC, MPD). All signals were recorded in the Time-Correlated Single Photon Counting (TCSPC) mode with a data acquisition board (TimeHarp 260, Picoquant). The FRET efficiency of the TS module, μ, was calculated based on the lifetime of the donor molecule:

1 $$\mu =1-\frac{{\tau }_{AD}}{{\tau }_{D}}$$

where *τ*_*AD*_ is the fluorescence lifetime of the donor molecule in the presence of the acceptor, and *τ*_*D*_ is the fluorescence lifetime of the donor molecule without the acceptor. The value of *τ*_*AD*_/*τ*_*D*_ is obtained by fitting the decay curve in the software Symphotime (Picoquant)^31,32^. The tension force is calculated based on the calibration curve of the TSmod reported in ref.^10^.

### Scratch assay

A wound healing scratch motility assay was utilized to evaluate 2-dimensional cell motility^33^. In brief, cells were grown on 12-well plates, and a plastic tip was used to scratch a gap onto the cell layer. After incubation, the areas newly occupied with cells in the scratched zone were imaged and measured with Image J (National Institutes of Health, Maryland, USA).

### Western blot analysis

Cells were lysed in a radio-immunoprecipitation assay (RIPA) buffer. Isolated proteins were fractionated using 10% SDS gels and electro-transferred to polyvinylidene difluoride membranes (Millipore, Billerica, MA, USA). We used antibodies against vinculin, Snail (Cell Signaling, Danvers, MA, USA), and β-actin (Sigma). Protein levels were assayed using a SuperSignal west femto maximum sensitivity substrate (Thermo Fisher Scientific).

### Knockdown of vinculin by siRNA and transfection of Snail plasmid

MLO-A5 osteocyte cells were treated with siRNA specific to vinculin (Cat No. 4392420, siRNA s532593, Life Technologies). A negative siRNA (Silencer Select #1, Life Technologies) was used as a nonspecific control. Cells were transiently transfected with siRNA using Lipofectamine RNAiMAX (Life Technologies) in Opti-MEM I medium. For overexpressing *Snail* protein, TMD tumor cells were transfected with a plasmid consisting of *Snail* coding sequence (SnailHA_pcDNA3; Addgene, Cambridge, MA, USA).

### Statistical analysis

Three or four independent experiments were conducted and data were expressed as mean ± S.D. Statistical significance was evaluated using one-way analysis of variance (ANOVA). Post hoc statistical comparisons with control groups were performed using Bonferroni correction with statistical significance set at *p* < 0.05.

## Supplementary information

Supplementary information

## Data Availability

All data generated or analyzed during this study are included in this published article (and its Supplementary Information files).
