# Supplementary material for: Vinculin Force Sensor Detects Tumor-Osteocyte Interactions
Source: Sci Rep. 2019 Apr 4;9:5615. doi: 10.1038/s41598-019-42132-x (PMC6449341; doi:10.1038/s41598-019-42132-x)
Supplement: Supplementary file 1 — Supplementary information [file 41598_2019_42132_MOESM1_ESM.pdf]

Supplementary Information

Vinculin Force Sensor Detects Tumor-Osteocyte Interactions

Fangjia Li, Andy Chen, Andrew Reeser, Yue Wang, Yao Fan[2], Shengzhi Liu,
Xinyu Zhao[2], Rahul Prakash, Divya Kota, Bai-Yan Li, Hiroki Yokota, and Jing Liu

1. Functional validation of the vinculin tension sensor in live TMD cells.

To validate the function of the VinTS in detecting molecular forces in focal adhesion, we evaluated the focal adhesion force when myosin activity was promoted or inhibited. In our experiments, live TMD cells were treated with ML-7, a myosin II inhibitor, and calyculin A, a myosin II activator. The inhibition or activation of the myosin II is expected to lead to reduced or increased tensions in live cells[1-3]. The FRET images of TMD cells show that ML-7 treated cells have higher FRET efficiency, i.e., smaller forces, in the focal adhesion sites than the control cells. Furthermore, the cells treated with calyculin A have lower FRET efficiency, i.e., higher forces. The histogram of FRET efficiency for each of the cells in Fig. S1A is plotted in Fig. S1B.

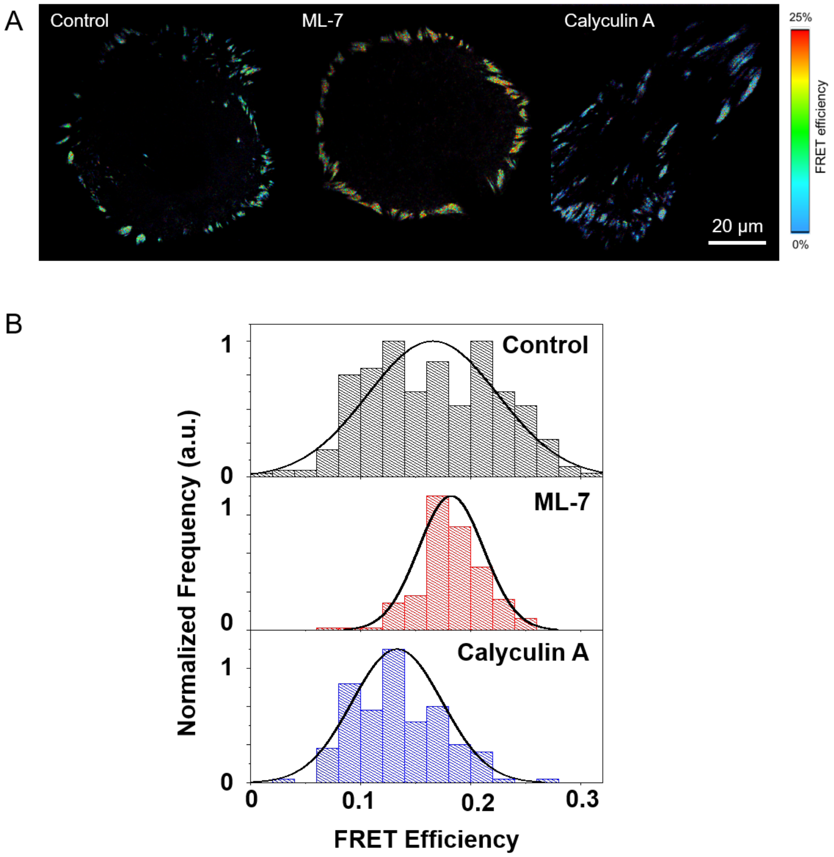

Suppl. Fig. S1. Validation experiment of the vinculin tension sensor. (A) Representative images, showing differences in FRET efficiency caused by the myosin inhibitor (ML7) and its activator (calyculin A). (B) Histograms of the distribution of FRET efficiency for control cells and cells treated with the inhibitor and activator.

2. Vinculin silencing leads to enhanced cell migration.

It is reported that vinculin acts as a retarder in cellular migration, and reduction in vinculin in tumor cells enhances their migratory behaviors.[4,5] To confirm this role of vinculin, we evaluated migratory behaviors of TMD tumor cells using the scratch assay. The result in Fig. S2 shows that the assay with vinculin-silenced tumor cells have a smaller wound area after 24 h. The relative wound area is shown in Fig. S2B.

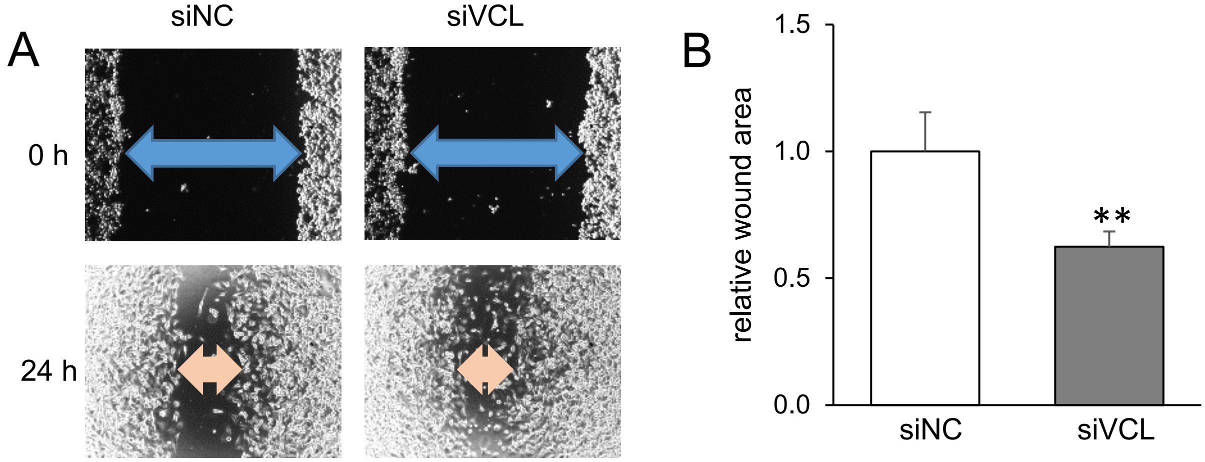

Suppl. Fig. S2. Cellular migration in a scratch assay of TMD cells, in which vinculin is partially silenced with siRNA. The double asterisk indicates $p < 0.01$.

3. Numerical simulation of tumor cell migration.

In parallel to experimental evaluation of the tumor cellular migration, we conducted numerical simulation. Based on the diffusion coefficient (slope of the MSD [mean square displacement] curve in Fig. 5), we simulated the trajectory of the migrating cells with a Brownian motion model (Fig. S3). The 24 h trajectory of a single TMD cell suggests random walking when TMD cell is migrating in the absence of osteocytes (Fig. S3A); the calculated MSD, based on the simulated trajectory, is nearly proportional to migration time, and its cumulative distribution function at 24 h follows Rayleigh distribution. The experimental and numerical results suggests that tumor cells exhibit random walking in the absence of osteocytes. As a comparison, tumor cells co-cultured with osteocytes suggest different migratory behaviors. As shown in Fig. S3B, migratory trajectories in the presence of a single osteocyte exhibit slower migration when a Brownian motion model is applied. Of note, the cumulative distribution function at 24 h does not follow Rayleigh distribution.

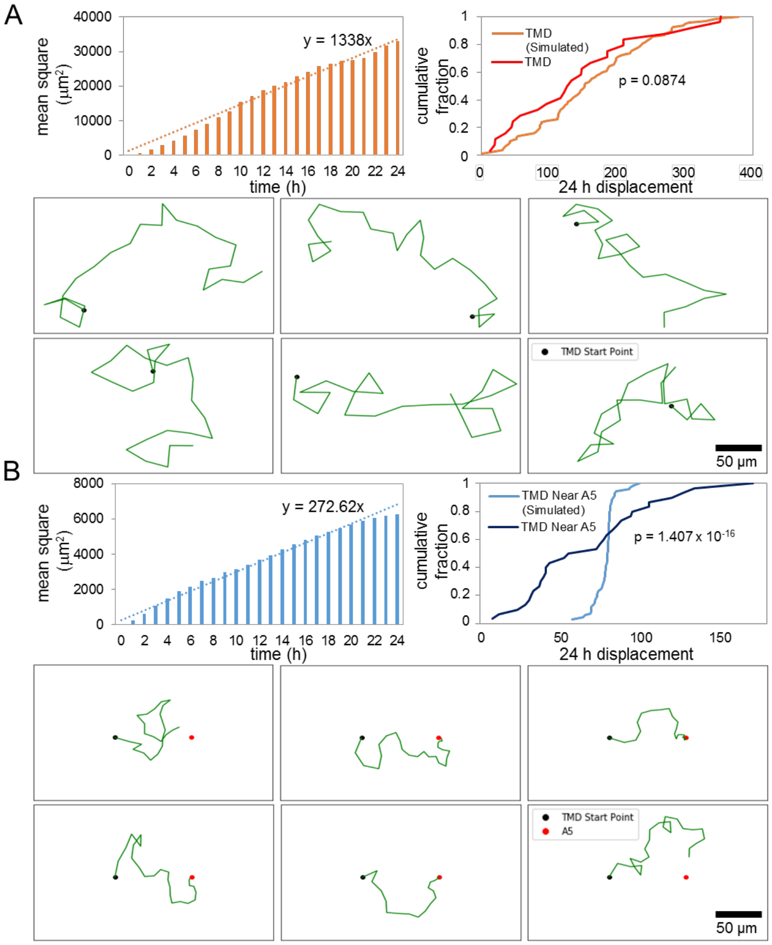

Suppl. Fig. S3. Numerical simulation was conducted to generate migratory trajectories of tumor cells using a random-walk model. (A) Migratory trajectories in the absence of osteocytes with a fixed step size of 24 μm. The mean-squared distance is nearly proportional to migration time, and its cumulative distribution function at 24 h follows Rayleigh distribution. (B) Migratory trajectories in the presence of a single osteocyte, which is located 80 μm from a tumor cell. The mean-squared distance is nearly proportional to migration time. Its cumulative distribution function at 24 h does not follow Rayleigh, as an exclusive subset of tumor cells are represented by this simulation.

4. Full images of the western blot results shown in the main manuscript.

The vinculin tension sensor is isolated from a plasmid (#26019, 4,764 bp insert in pcDNA3.1; Addgene), with a molecular mass of ~175 kDa. The expected molecular mass of endogenous vinculin is 124 kDa. In Western blot analysis (Fig. S4A), we detected endogenous vinculin and confirmed its downregulation by vinculin specific siRNA. We did not detect the vinculin tension sensor (TS) because of its low expression level. Collectively, the result validated that transfection of vinculin TS did not elevate an overall concentration of vinculin.

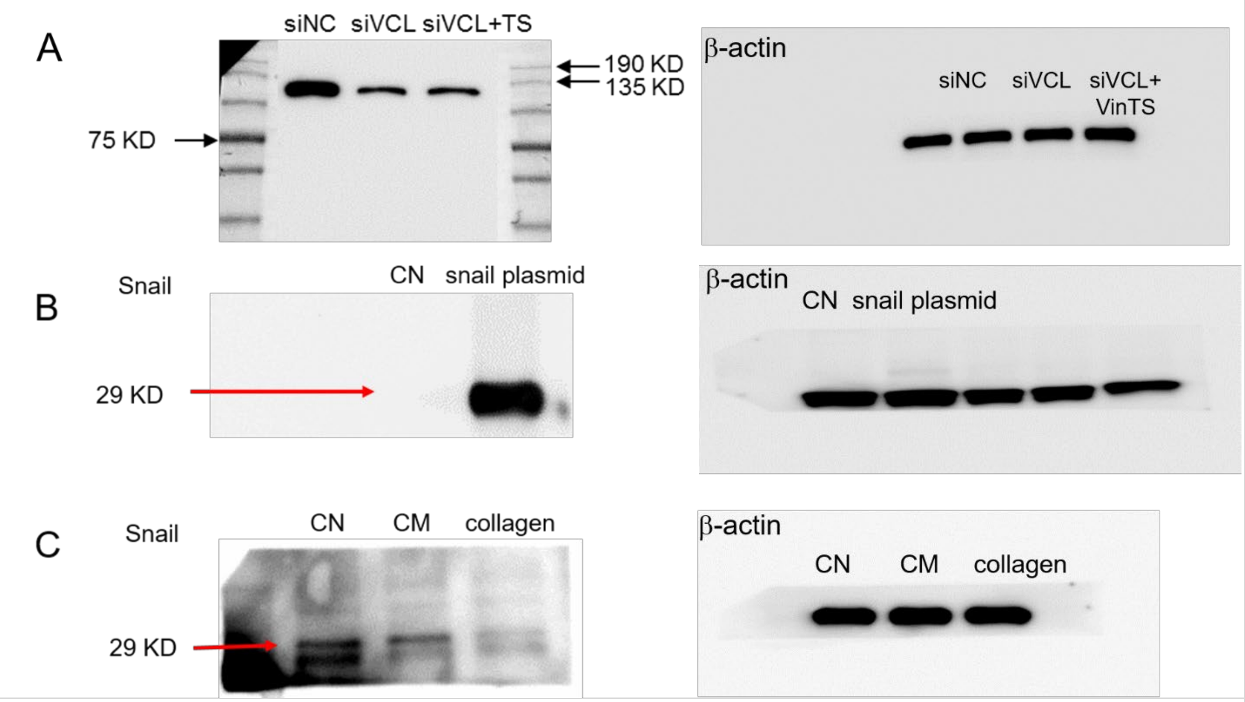

Suppl. Fig. S4. Full length images of the gels and western blots in Figure 1D (A, B) and Figure 4D (C).

References:

1. Watanabe, T., Hosoya, H., and Yonemura, S., Regulation of myosin II dynamics by phosphorylation and dephosphorylation of its light chain in epithelial cells. Molecular Biology of the Cell, 18, 605-616 (2007).
2. Mierke, C., et al. Focal adhesion kinase activity is required for actomyosin contractility-based invasion of cells into dense 3D matrices. Scientific Reports, 7, 42780 (2017).
3. Suzuki, A., and Itoh, T., Effects of calyculin A on tension and myosin phosphorylation in skinned smooth muscle of the rabbit mesenteric artery. Br. J. Pharmacol. 109, 703-712 (1993).
4. Toma-Jonik, A. *et al.* Active heat shock transcription factor 1 supports migration of the melanoma cells via vinculin down-regulation. *Cell. Signal.* **27,** 394–401 (2015).
5. 20. Dumbauld, D. W. *et al.* How vinculin regulates force transmission. *Proc. Natl. Acad. Sci.* **110,** 9788–9793 (2013).
